# A Preliminary Study on Photic Driving in the Electroencephalogram of Children with Autism across a Wide Cognitive and Behavioral Range

**DOI:** 10.3390/jcm11133568

**Published:** 2022-06-21

**Authors:** Luigi Vetri, Laura Maniscalco, Paola Diana, Marco Guidotti, Domenica Matranga, Frédérique Bonnet-Brilhault, Gabriele Tripi

**Affiliations:** 1 Oasi Research Institute-IRCCS, 94018 Troina, Italy; lvetri@oasi.en.it; 2 Department of Health Promotion, Mother and Child Care, Internal Medicine and Medical Specialties, University of Palermo, 90127 Palermo, Italy; laura.maniscalco04@unipa.it (L.M.); gabriele.tripi@unipa.it (G.T.); 3 Department of Neuropsychiatry of Childhood and Adolescence, S. Marta and S. Venera Hospital, ASP Catania, 95024 Catania, Italy; diana.pao@hotmail.it; 4 UMR1253, iBrain, University of Tours, INSERM, 37000 Tours, France; marco.guidotti@univ-tours.fr (M.G.); frederique.brilhault@univ-tours.fr (F.B.-B.); 5 Excellence Center in Autism and Neurodevelopmental Disorders, Centre Hospitalier Régional Universitaire, 37000 Tours, France

**Keywords:** autism spectrum disorder, intermittent photic stimulation, electroencephalography

## Abstract

Intermittent photic stimulation (IPS) is a useful technique in electroencephalography (EEG) to investigate the neurophysiological anomalies of brain activity. Although not an active task, IPS has also been explored in ASD; it is thought to capture local potential oscillators at specific frequencies and perhaps tap into rhythmic activity in a way that general resting-state recordings cannot. Previous studies suggest that individuals with ASD showed photic driving reactivity predominantly at lower frequencies of stimulation. In our study we used IPS to measure rhythmic oscillatory activity in a sample of 81 ASD children. We found a significant correlation linking ASD children with photic driving activation only at low frequencies (δθ band) and increased severity of “restricted behavior”. This suggests that ASD children with higher severity of restricted behaviors could have a hypersynchronous θ power and an impaired resonance synchronization at middle-ranged frequencies (α). Furthermore, we found some evidence of hemispherical oscillatory asymmetry linked particularly to behavioral impairments. This result is in line with the EEG pattern model indicating a “U-shaped profile” of electrophysiological power alterations with excess power in low- and high-frequency bands and a reduction of power in the middle-ranged frequencies. IPS technique in electroencephalography is confirmed to reveal EEG biomarkers in autistic children, with a focus on spectral power, coherence, and hemisphere asymmetries.

## 1. Introduction

Autism spectrum disorder (ASD) is one of the most common developmental disabilities characterized by a deficit in social skills and by the presence of repetitive behaviors and/or restricted interests [1].

Abundant literature suggests that many neurophysiological pathways are involved in determining ASD. Theoretically, disruptions in synaptogenesis, pruning, and myelination lead to an array of atypical neural networks that then manifest as a very recognizable behavioral phenotype [2,3].

Careful analysis of electrophysiological patterns across the brain using electroencephalogram (EEG) coherence is one way to evaluate this synaptic dysfunction in a noninvasive manner.

In electroencephalography, the activation procedures or functional tests enhance the manifestation of latent neurophysiological mechanisms or pre-existing abnormalities and may induce abnormal findings in an otherwise normal EEG [4]. Intermittent photic stimulation (IPS) is commonly used in current electroencephalography to test some basic neurophysiological mechanisms, taking into account modern concepts of the organizing role of the bioelectrical rhythms in the brain [5].

IPS can induce, in the EEG, photic driving (PD), consisting of synchronous rhythmic activity to the light stimulus at the same frequency or harmonically related to the frequency of the stimulus. In the EEG spectrogram, driving appears as sharp amplitude peaks at the stimulation frequency and its harmonics [6].

Different individual PD profiles seem to exist, but for each subject the PD profile seems to be always the same over time [7,8], and it is more frequent during childhood than adulthood [4,9,10]. A greater increase in amplitude is found during photic stimulation near alpha frequencies of the background EEG [10,11,12]. A PD reaction to the theta band is a characteristic of the prepubertal and pubertal periods and there is no literature evidence of theta synchronization in healthy adults [10]. On the other hand, the absence of PD reaction has reduced clinical significance because some normal individuals do not show responsiveness to IPS [13]. Nevertheless, the parameters of the driving response may serve as sensitive EEG indicators of the peculiarities of age and brain maturation [4,14].

To date, few previous studies have investigated the use of IPS to measure rhythmic oscillatory activity in ASD subjects [15,16,17]. These authors found that the ability to phase lock alpha and beta oscillations to repetitive stimulation is reduced in ASD, suggesting that synchronization of neural assemblies at moderate frequencies in response to sensory inputs is impaired in ASD.

On the other hand, these studies have focused on the individuals with cognitive abilities in the typical range, thus excluding the large portion of the ASD population with co-occurring cognitive impairment. Moreover, these studies often highlight prespecified, putative networks of interest, such as brain regions involved in language function or social cognition. Such a targeted approach could preclude an investigation on global autistic behavior and differences in meaningful circuits. It is thus reasonable to conjecture that electroencephalography (EEG) signals may demonstrate discernible patterns, reflecting information about the underlying neural networks that anticipate changes in behavior [18].

This preliminary research aims to provide further evidence that IPS may be highly beneficial in revealing latent differences between ASD groups and cognitive and behavioral features that are unobservable in spontaneous resting-state EEG.

To our best knowledge, this might be one of the first studies combining oscillatory reactions to IPS at different fixed frequencies and cognitive and behavioral assessment in children with ASD.

## 2. Materials and Methods

This is a retrospective study based on clinical data, including neurophysiological, behavioral, and cognitive assessments, which were collected from medical records and stored in a bioclinical database at the Excellence Center for Autism—Tours. Informed consent was obtained from all subjects involved in the study. The study was conducted in accordance with the Declaration of Helsinki and approved by CNIL, the French data protection authority at the Excellence Center for Autism—Tours). Ethical approval was obtained from the Ethics Committee of the Tours University Hospital (no. 2020 074).

### 2.1. Participants

Patients were fully assessed by a specialized, multidisciplinary team of the Excellence Center for Autism—Tours. The sample consisted of 81 patients (67 males and 14 females; aged 3–16 years at the time of electroencephalographic exam) with a diagnosis of ASD according to ICD-10 criteria. In particular, the sample of ASD children included those who met diagnostic criteria for childhood autism (F84.0—45 patients), atypical autism (F84.1—8 patients), Asperger’s syndrome (F84.5—3 patients), other pervasive developmental disorders (F84.8—12 patients), and pervasive developmental disorder, unspecified (F84.9—13 patients) (Table 1). ASD was diagnosed using standardized diagnostic tools such as the Autism Diagnostic Observation Schedule Second Edition (ADOS) [19], a semi-structured assessment, and the Autism Diagnostic Interview-Revised (ADI-R) [20], a structured interview for parents. All patients at the time of assessment did not show epileptic seizures or EEG abnormalities. They were free of drug treatment. A synthesis of demographic and clinical characteristics of the sample is shown in Table 1.

### 2.2. Cognitive and Behavioral Assessment

Cognitive and language assessments were tailored to the ability and age of the child, and ratio IQ was used to facilitate comparison across assessments.

The tests used were the following: Wechsler Preschool and Primary Scale of Intelligence-Third Edition (WPPSI-III) [21] and Wechsler Intelligence Scale for Children (WISC-IV) [22], Échelles Différentialles d’Efficience Intellectuelle-forme révisée (EDEI-R) [23]. For children who were assessed with the Wechsler’s tools, NVIQ and VIQ were calculated from the protocol-specific subscores. EDEI-R scales were elaborated for atypical populations and their applicability to subjects with intellectual deficits was confirmed [24]. These scales allow distinguishing the verbal developmental age (VDA) and the nonverbal developmental age (NVDA). The VDA was calculated by means of the scores obtained on five scales: vocabulary as pictures denomination, vocabulary as word definition, knowledge, social understanding, and conceptualization. The NVDA was calculated by means of the scores obtained on four scales: classification of couples of pictures, classification of three pictures, categorial analysis, and practical adaptation.

Behavioral assessment was carried out using the Childhood Autism Rating Scale (CARS) [25] and the Repetitive and Restricted Behaviors scale (RRB scale) [26].

The CARS evaluates the severity of autistic behaviors in 14 functional areas by assigning a score from 1 to 4. An overall score is calculated by adding scores from all functional areas. It enables the assessment of severity of autistic symptoms, and the stratification of patients into three levels: “severely autistic” (score between 37 and 60), “mildly to moderately autistic” (score between 30 and 36.5), and “absence of ASD” (score of less than 30). The questionnaire can be carried out in around 20–30 min.

The RRB scale is a valid tool to describe four specific meaningful factors within the repetitive and restricted behaviors of ASD: factor 1: sensorimotor stereotypies (RRB-F1); factor 2: reaction to change (RRB-F2); factor 3: restricted behaviors (RRB-F3); and factor 4: modulation insufficiency (RRB-F4). The RRB scale comprises 35 items evaluated according to a five-level Likert scale (0 = “the behavior is never expressed by the person”, 1 = “weakly expressed”, 2 = “moderately expressed”, 3 = “severely expressed”, and 4 = “the behavior is very characteristic of the person and very severely expressed”).

At the end of the multidisciplinary assessment, the team was able to assign a score to the two symptom dimensions, social communication/interaction, and restricted/repetitive behaviors (SCI and RRB), according to DSM-5.

### 2.3. EEG Recording

EEG signal acquisition was made through Deltamed-Coherence 5.1 system (Natus Medical Incorporated, San Carlos, CA, USA). The scalp electrodes were placed according to 10–20 international system thanks to Electrocap^®^ EEG headset (Electro-Cap Center B.V. The Netherlands) (analog low-pass filter 1500 Hz at −3 dB). The EEG sampling frequency was 256 Hz. A digital low-pass filter was set at 1/3 of sampling frequency. During EEG visualization, a time constant of 0.3 s was set corresponding to a high-pass filter of 0.5 Hz at −3 dB and a low-pass filter of 35 Hz.

The recording included 10 min of quiet wakefulness without any activation procedures followed by an IPS of the total duration of 230 s, 10 s for each frequency. IPS frequency increased gradually from 1 to 30 Hz (ascending stimulation) then decreased gradually in the same way from 30 to 1 Hz (descending stimulation) as follows: 1, 3, 5, 7, 10, 12, 15, 17, 20, 22, 25, 30, 25, 22, 20, 17, 15, 12, 10, 7, 5, 3, 1 Hz.

**Figure 1:**
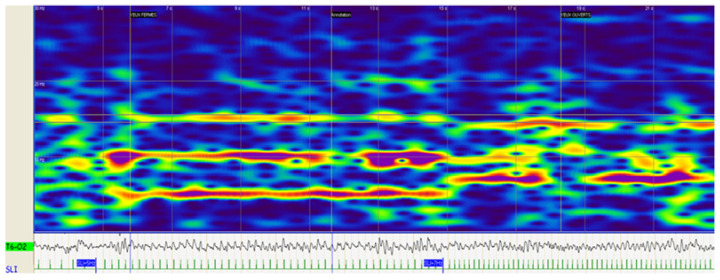
Time–frequency analysis through continuous wavelet transform.

A time–frequency analysis using continuous wavelet transform was performed on the derivations T5-O1 of the left hemisphere and on T6-O2 of the right hemisphere within the 0–30 Hz EEG frequency band. This analysis allowed us, for any IPS frequency, to determine the presence or absence of a PD response (Figure 1). The frequency of 1 Hz was not studied because the wavelet analysis could be altered in very low frequencies by a patient’s movement or electrode drift. In the same way, the frequency of 30 Hz was not considered because there were no patients that showed a PD response at this frequency.

Each signal or time–frequency was decomposed into three power-of-two frequency bands that are approximately equal to the commonly used EEG frequency band labels (Table 2). For each power frequency; the PD response was considered as present when it occurred in the two frequencies of stimulation, and as absent when it occurred in only one frequency of stimulation or if there was not any PD synchronization.

### 2.4. Statistical Analysis

The EEG data of 81 patients were analyzed by multivariate statistical analysis that considered 12 binary variables: presence/absence of a PD synchronization for each of the three EEG bands, for ascending and descending stimulation, and for right and left hemispheres. These 12 qualitative EEG variables were first factor-analyzed using multiple correspondence analysis (MCA). Similar to what is performed with principal component analysis, this analysis identified the main factors that were entered into a cluster analysis, which classifies patients according to the similarity of their profiles. Specifically, an agglomerative hierarchical clustering (Ward method) was processed using Euclidean distance.

Then, a discriminant analysis on the 12 EEG variables was performed to verify the robustness of the results obtained.

Nonparametric statistical analyses (χ^2^ test, Kruskal–Wallis H- and Mann–Whitney U-tests) were performed to compare groups of patients described by cluster analysis according to gender, age, verbal and nonverbal IQ scores, CARS scores, RRB scores (F1, F2, F3, F4), and impairment for two symptoms according to DSM-5 (SCI and RRB).

McNemar’s test was applied to assess if there was a statistically significant difference in three frequency bands of IPS of the right compared to the left hemisphere, both in ascending and descending stimulation.

Afterwards, linear models were applied to assess if demographic characteristics, verbal and nonverbal IQ scores, expressive and receptive language, RRB scores, DSM-5, and CARS scores were correlated with the difference between the right and left hemispheres both for the ascending and descending IPS frequencies. Statistical analysis was performed using Statistica V12 software (TIBCO Statistica, Palo Alto, CA, USA) and STATA/SE 14.

In all cases, tests were performed on the two-sided 5% level of significance.

## 3. Results

MCA carried out on the 12 EEG binary variables allowed us to identify three factors accounting for 72.0% of total variance (F1: 44.8%, F2: 17.1%, F3: 10.1%). Then, cluster analysis performed on these three factors allowed us to retain a four-cluster solution. This solution was confirmed by a discriminant analysis on the original 12 EEG variables, underlining that 97.5% of patients had been correctly classified. Therefore, four groups of patients according to PD synchronization to three frequency bands and for ascending and descending IPS on both hemispheres were selected; PD δθ/α +: group of 32 ASD children showing PD synchronization at delta–theta and alpha frequency bands; PD −: group of 25 ASD children without PD synchronization during IPS; PD δθ+: group of 14 ASD children with PD synchronization at delta–theta frequency band; PD δθ/α/β +: group of 10 ASD children with PD synchronization at all three frequency bands. A significant association was found between PD δθ+ group and RRB-F3 score (restricted behavior). Post hoc analysis showed that this feature was linked to more severe restricted behaviors in the PD δθ+ group than in the PD δθ/α/β + group. Any other difference between the four groups was not found (Table 3).

McNemar’s test showed that there were not statistically significant differences for IPS frequencies response between the right and the left hemispheres, both for ascending (*p*-value > 0.05) and for descending (*p*-value > 0.05) stimulation.

Hemispheric differences between EEG powers and both cognitive and behavioral features were assessed. The linear models highlighted that among the V/NVIQ scores, children having PD in the β frequency band at IPS descending phase only in the left hemisphere showed a higher VIQ (beta = 62.65, 95% CI = 9.08–116.23, *p*-value = 0.023). On the other hand, ASD children with PD at δθ frequencies band at IPS only on the left hemisphere showed lower NVIQ scores compared to those who do not have differences between left and right hemisphere (beta = −31.29, 95% CI = −57.89–−4.68, *p*-value = 0.022).

Among the RRB scores, the ASD children with PD in δθ frequency band in IPS descending phase on the left hemisphere had higher probability to have sensorimotor stereotypies (RRB-F1 score) compared to children that do not have differences between the left and the right hemisphere (beta = 6.97, 95% CI = 1.03–12.91, *p*-value = 0.022), while ASD children with PD at δθ frequency in IPS descending phase only in the right hemisphere had a higher modulation insufficiency (RRB-F4 score) compared to children that do not have differences between the left and the right hemisphere (beta = 11.04, 95% CI = 6.84–15.23, *p*-value < 0.001).

## 4. Discussion

Many studies suggest that ASD is a connectivity disorder [27]. Electroencephalography, which primarily measures neurophysiological changes related to postsynaptic activity in the neocortex [26], has proven to be a powerful tool for studying complex neuropsychiatric disorders [28,29]. It is thus reasonable to conjecture that EEG investigations in different power bands and coherence between hemispheres and brain regions may demonstrate discernible patterns, reflecting information about the underlying neural networks that highlight changes in intellectual and behavioral ASD impairments. The literature on the EEG powers correlated in ASD is quite discrepant and usually describes general and nonspecific changes, such as some prevalence of asymmetrical bioelectrical activity and rhythms suppression and disorganization [16,17,30].

EEG intermittent photic stimulation, although not an active task, has also been explored in ASD; it is thought to capture local potential oscillators at specific frequencies and perhaps tap into rhythmic activity in a way that general resting-state recordings cannot [17].

In a previous study, Lazarev and colleagues [16,17] measured activity only in 14 relatively homogeneous subjects with ASD, revealing that the ability to phase lock α and β oscillations to repetitive visual stimulation is reduced in ASD. Collectively, these studies led to the conclusion that synchronization of neural assemblies at moderate frequencies in response to sensory inputs is impaired in ASD [31].

On the other hand, these studies did not conduct correlational analyses to relate abnormal EEG patterns to severity of various clinical aspects of ASD. This limits the comprehension of the clinical relevance of IPS in EEG observations.

This preliminary research shows that in our sample of ASD children, across a wide cognitive and behavioral range, there coexist heterogeneous PD responses to IPS. According to our results, about 30% of patients do not show a PD response at all frequencies. This feature is nearly matched with that of the general population, according to the literature data [10,32].

Most patients showed an age-appropriate IPS response profile, with the ability to phase lock in the low frequencies (as occurs in healthy subjects during childhood and adolescence), and in the middle-ranged/high frequencies (usually at the same EEG background frequency) to repetitive visual stimulation [10,11,12]. These ASD children did not show any relationship with demographic, cognitive, and behavioral features.

On the other hand, interestingly, our results showed a link between ASD children with PD activation only at low frequencies (δθ band) and increased scores for the RRB factor of “restricted behavior”. This behavioral dimension reflects for this ASD group higher difficulty dealing with change and preference for routines. This could suggest that ASD children with higher severity of restricted behaviors could have an impaired resonance synchronization at middle-ranged frequencies (α) and high frequencies (β).

This finding is in line with Cohen et al. [30], who found increase of θ frequency, especially over the right posterior region. Cohen and his coauthors also found a related reduction in high frequencies over the right hemisphere for the ASD group.

The exact role of θ activity in clinical populations is not fully understood; excess of hypersynchronous θ power is not specific for autism and is commonly found in children with executive functioning and mental activity problems, including attention deficit/hyperactivity disorder [32], learning disabilities [33], and intellectual disability [34]. Given the high overlapping between autism and these neuropsychiatric disorders, evidence of hypersynchronous θ power in autism might be related to some specific genetic etiology of autism or comorbidity [35].

Moreover, this finding is coherent with evidence of altered α recruitment in ASD visual processing when using photic driving [36]. In addition to abnormalities in visual-stimulus-induced α activity, there is also evidence that top-down modulation of α power is impaired in ASD, and these differences have been robustly linked with behavioral impairments. Children with ASD fail to appropriately modulate α power when performing a version of attentive task with competing auditory and visual stimuli [37]. Furthermore, as oscillations have been strongly tied to thalamo-cortical interactions [38,39], α dysfunction suggests that changes in subcortical function also contribute to the atypical oscillatory function observed in ASD.

Previous studies have also showed that an inhibitory interneuron impairment is the main neurophysiological mechanism causing a reduced spontaneous EEG synchronous oscillation in children with autism. These studies showed impairment in right-hemisphere reactivity, suggesting a hemispherical oscillatory asymmetry. This could be related to a decrease of the deep white matter in the right hemisphere of autistics observed in neuroimaging studies [15,35].

Chan and colleagues [40] demonstrated a significantly lower relative α and higher relative δ and δ–α ratio in ASD individuals, but the authors considered that such abnormality in relative α power among ASD children is not restricted only to a single and specific location of the brain but, on the contrary, is a widespread pattern across the brain, possibly reflecting the neurophysiological abnormality associated with ASD.

Our results did not show any hemispheric asymmetry about power bands between right and left hemispheres. On the other hand, we found some evidence of hemispheric oscillatory asymmetries (particularly in the δθ band) linked to the behavioral factors of sensorimotor stereotypies and modulation insufficiency as well as in verbal/nonverbal cognitive skills.

In the literature, sensorimotor stereotypies appear to be mainly associated with anxiety and severity of core autism symptoms [41], while the restricted behaviors and modulation insufficiency seem to constitute two more complex behavioral dimensions related to autism but also influenced by intellectual disability or other comorbid medical and psychiatric condition [26].

Only few studies report correlational analyses relating frequencies powers to severity of various cognitive and behavioral aspects of ASD: Orekhova et al. [42] found a positive correlation between high frequencies (gamma) activity and cognitive delay in ASD. Sutton et al. [43] reported that abnormal left frontal asymmetry, defined by greater activation in the left frontal regions, was related to higher levels of social anxiety and social stress, as was abnormal right frontal asymmetry. Stroganova et al. [35] showed that increased prefrontal δ power was related to cognitive delay in ASD. Burnett et al. [44] reported an association of left frontal EEG asymmetry with parental reports of later onset of ASD symptoms, and increased instances of aggressive outbursts and obsessive–compulsive behavior. Finally, Brugger et al. [45] proposed the suppressed activity in regions of the right hemisphere underlying the prominent piecemeal processing as well as repetitive stereotyped behavior in ASD. Thus, rightward asymmetry has been explicitly hypothesized in the literature to be a fundamental characteristic of cerebral organization in ASD.

The interaction of ASD, intellectual disability, and other comorbidities led to the hypothesis of general dysregulation of E/I balance, caused by defects in GABAergic fibers, particularly GABAergic interneurons maturation, or GABA receptor function [46]. In their review of resting-state EEG studies in ASD, Wang and colleagues [29] reported a potential “U-shaped” profile of EEG power spectra in ASD as compared to typically developing controls, with excess power in low- and high-frequency bands and decreased power in middle-ranged frequency band. This resting EEG profile highlights the hypothesis that ASD oscillatory dysfunctions could be attributed to affected GABAergic interneurons that have a modulating role on excitatory pyramidal cells. Although Wang and colleagues [29] identify this U-shaped profile as an EEG biomarker observed in resting state, on the contrary, a recent comprehensive review [47] underscores that no general pattern can be inferred within EEG findings among patients with ASD. On the other hand, interestingly, this “U-shaped” profile in a resting condition is in line with our preliminary findings based on repetitive visual stimulation technique, representing a potential biomarker of disrupted oscillatory synchronization in ASD. Indeed, as a recent work highlights [48], the use of visual stimuli could have a particular impact on α power, suggesting that resting conditions with visual input elicit reduced α power compared to resting conditions without such input.

In addition, in postmortem brain samples of ASD cases, a reduction was found of neocortical minicolumns, elemental modular microcircuits made up of excitatory pyramidal neurons surrounded by GABAergic inhibitory neurons, which could result in inhibitory circuits disruption [49]. Defects in GABAergic signaling, especially shifting the E/I balance toward excitatory transmission, may thereby explain some of the characteristics of oscillatory impairment in ASD with wide behavioral and cognitive dimensions [50].

### Limitations and Implications for Treatment Research

Our results support the hypothesis of previous studies that an altered ratio of connectivity in theta and alpha oscillatory activity may be characteristic of ASD. Despite the larger sample size of our research compared to these previous studies, the lack of a control group of children with typical neurodevelopment is the primary limitation of our study. Furthermore, the uneven distribution of children by age makes the comparison between ASD groups and clinical features difficult, given the extensive variation in neuromaturation that occurs during the first 25 years of life [51].

Typical development is characterized by two major age-dependent changes in neural oscillatory powers, a decrease in slow oscillatory powers (δ, θ, and α) mainly in the caudal region, and an increase in the fast oscillatory powers (β and γ) mainly in the rostral region [48,52]. One three-month longitudinal study [42] in ASD children indicated that EEG characteristics are relatively stable across short time intervals. This suggests that the developmental aspect of oscillatory regulation deserves special investigation with age-matched longitudinal studies to understand whether individuals with ASD show similar trajectories of functional connectivity maturation, or whether these processes are disrupted and/or delayed. Similarly, gender differences in the developmental trajectories of oscillatory power are important to consider. The literature highlights that, in healthy subjects, one prominent structural gender difference in oscillatory powers is found in a broad frequency range, mostly in the caudal brain regions [52]; males show stronger power in lower frequencies in the left caudal region while females show stronger power in higher frequencies in the right caudal region. Our findings did not show any correlation between power frequencies and gender differences in ASD children. Thus, developmental variations in power at different frequencies, coherence, and functional lateralization are important considerations when studying developmental disorders such as ASD, in which behavioral and cognitive presentations are different for males and females and can change over the age span.

Another potential limitation of this study could be the important variability of ASD phenotype in our sample, including comorbid medical and psychiatric conditions. In this case, IPS could be even more informative for diagnostics because it shows its potential in revealing different neurophysiological aspects of latent functional pathology not present in the spontaneous EEG of the resting state [31]. Thus, it might become increasingly important to subgroup or control for the different conditions that are grouped under the ASD umbrella to better understand the impact of this heterogeneity on the nature of EEG oscillatory dysfunctions in ASD.

## 5. Conclusions

The application of IPS is confirmed as a technique to focus on the dynamic function of the ASD brain as well as on a more precise definition of clinical subtypes correlated to specific neurophysiological and genetic profiles, in order to provide ever more personalized and targeted treatments. We strongly believe that combining IPS techniques in EEG resting-state studies could potentially open a new perspective on ASD assessment and eventually lead to early diagnosis, early intervention, and prevention strategies.

## Figures and Tables

**Table 1:** Demographic and clinical characteristics of the sample (*n* = 81).

	*n* (%)
Male	67 (82.72%)
Female	14 (17.28%)
Diagnosis	*n* (%)
F84.0	45 (55.56%)
F84.1	8 (9.88%)
F84.5	3 (3.7%)
F84.8	12 (14.81%)
F84.9	13 (16.05%)
IQ	*n* (%)
>70	42 (51.85%)
69–50	22 (27.16%)
49–35	12 (14.82%)
34–20	5 (6.17%)

**Table 2:** Correspondence between wavelet frequency range and standard EEG frequency band labels.

Wavelet Frequency Range	Approximate EEG Labels
3–7 Hz	Delta–Thêta (δθ)
10–15 Hz	Alpha (α)
17–25 Hz	Beta (β)

**Table 3:** Correlations between ASD groups and demographic, cognitive, and behavioral data.

	PD δθ/α+ N./Mean/(sd)	PD − N./Mean/(sd)	PD δθ+ N./Mean/(sd)	PD δθ/α/β + N./Mean/(sd)	ANOVA *p*-Value	Post hoc Analysis
AGE (MONTHS)	32/110.6/(41.5)	25/108.4/(42.7)	14/93.9/(42.2)	10/118.9/(51.3)	0.509	/
VIQ	23/75.0/(33.3)	21/59.9/(35.6)	13/61.3/(33.9)	8/59.0/(41.6)	0.334	/
NVIQ	22/83.9/(28.7)	21/73.9/(36.7)	13/81.8/(24.9)	8/65.7/(23.4)	0.380	/
CARS	29/29.1/(7.1)	22/31.1/(6.1)	13/29.2/(5.3)	9/27.5/(3.8)	0.401	/
RRB-F1	28/6.3/(6.5)	21/6.2/(6.0)	14/9.7/(9.4)	10/5.4/(5.6)	0.682	/
RRB-F2	28/2.4/(2.8)	21/2.6/(3.5)	14/3.5/(4.9)	10/1.8/(2.0)	0.972	/
RRB-F3	28/4.6/(3.5)	21/5.4/(5.2)	14/7.4/(5.7)	10/2.2/(1.9)	**0.044**	PD δθ+ > PD δθ/α/β +
RRB-F4	28/4.1/(3.4)	21/3.9/(2.8)	14/5.2/(3.1)	10/4.9/(2.5)	0.450	/
DSM5-SCI	25/1.5/(0.7)	17/1.6/(0.7)	11/1.4/(0.5)	8/1.0/(0.0)	0.147	/
DSM5-RRB	25/1.4/(0.6)	17/1.7/(0.8)	11/1.9/(0.8)	8/1.4/(0.5)	0.194	/

SD: standard deviation; VIQ: verbal intelligence quotient; NVIQ: nonverbal intelligence quotient; CARS: Childhood Autism Rating Scale; RRB: Repetitive and Restricted Behaviors scale—scale of restricted and repetitive behaviors; SCI: social communication/interaction; RRB: restricted/repetitive behaviors; Statistically significant *p*-values are in bold.

## Data Availability

Data are available upon request by CNIL.

## References

[1] Edition F. (2013). Diagnostic and statistical manual of mental disorders. Am. Psychiatr. Assoc..

[2] Zikopoulos B., Liu X., Tepe J., Trutzer I., John Y.J., Barbas H. (2018). Opposite development of short-and long-range anterior cingulate pathways in autism. Acta Neuropathol..

[3] Geschwind D.H., Levitt P. (2007). Autism spectrum disorders: Developmental disconnection syndromes. Curr. Opin. Neurobiol..

[4] Niedermeyer E., da Silva F.H.L. (2005). Electroencephalography: Basic Principles, Clinical Applications, and Related Fields.

[5] Livanov M.N. (1977). Spatial Organization of Cerebral Processes.

[6] Chatrian G.E. (1974). A glossary of terms most commonly used by clinical electroencephalographers. Electroencephalogr. Clin. Neurophysiol..

[7] Tyler C.W., Apkarian P., Nakayama K. (1978). Multiple spatial-frequency tuning of electrical responses from human visual cortex. Exp. Brain Res..

[8] Fedotchev A.I., Bondar A.T., Konovalov V.F. (1990). Stability of resonance EEG reactions to flickering light in humans. Int. J. Psychophysiol..

[9] Beydoun A., Schechter S.H., Nasreddine W., Drury I. (1998). Responses to photic stimulation in patients with occipital spikes. Electroencephalogr. Clin. Neurophysiol..

[10] Lazarev V.V., Simpson D.M., Schubsky B.M., Deazevedo L.C. (2001). Photic driving in the electroencephalogram of children and adolescents: Harmonic structure and relation to the resting state. Braz. J. Med. Biol. Res..

[11] Walter V.J., Walter W.G. (1949). The central effects of rhythmic sensory stimulation. Electroencephalogr. Clin. Neurophysiol..

[12] Danilova N.N. (1985). Functional States: Mechanisms and Diagnostics.

[13] Kooi K.A., Tucker R.P., Marshall R.E. (1978). Fundamentals of Electroencephalography.

[14] Kaiser J., Gruzelier J.H. (1996). Timing of puberty and EEG coherence during photic stimulation. Int. J. Psychophysiol..

[15] Lazarev V.V., Pontes A., deAzevedo L.C. (2009). EEG photic driving: Right-hemisphere reactivity deficit in childhood autism. A pilot study. Int. J. Psychophysiol..

[16] Lazarev V.V., Pontes A., Mitrofanov A.A., DeAzevedo L.C. (2010). Interhemispheric asymmetry in EEG photic driving coherence in childhood autism. Clin. Neurophysiol..

[17] Lazarev V.V., Pontes A., Mitrofanov A.A., Deazevedo L.C. (2015). Reduced interhemispheric connectivity in childhood autism detected by electroencephalographic photic driving coherence. J. Autism Dev. Disord..

[18] Dickinson A., DiStefano C., Lin Y.-Y., Scheffler A.W., Senturk D., Jeste S.S. (2018). Interhemispheric alpha-band hypoconnectivity in children with autism spectrum disorder. Behav. Brain Res..

[19] Lord C., Risi S., Lambrecht L., Cook E.H., Leventhal B.L., DiLavore P.C., Pickles A., Rutter M. (2000). The Autism Diagnostic Observation Schedule—Generic: A standard measure of social and communication deficits associated with the spectrum of autism. J. Autism Dev. Disord..

[20] Lord C., Rutter M., Le Couteur A. (1994). Autism Diagnostic Interview-Revised: A revised version of a diagnostic interview for caregivers of individuals with possible pervasive developmental disorders. J. Autism Dev. Disord..

[21] Wechsler D. (2014). Echelle D’Intelligence de Wechsler Pour la Période Pré-Scolaire ET Primaire—Quatrième ÉDition (WPPSI-IV).

[22] Wechsler D. (2005). ÉChelle D’Intelligence de Wechsler Pour Enfants: WISC-IV.

[23] Perron-Borelli M. (1996). EDEI-R: Echelles Différentielles D’Efficiences Intellectuelle.

[24] Tourrette C. (2006). Evaluer les Enfants avec des Déficiences ou Troubles du Développement. [To Assess Children with Disabilities or Developmental Disorders].

[25] Schopler E., Reichler R.J., DeVellis R.F., Daly K. (1980). Toward objective classification of childhood autism: Childhood Autism Rating Scale (CARS). J. Autism Dev. Disord..

[26] Bourreau Y., Roux S., Gomot M., Bonnet-Brilhault F., Barthélémy C. (2009). Validation of the repetitive and restricted behaviour scale in autism spectrum disorders. Eur. Child Adolesc. Psychiatry.

[27] Assaf M., Jagannathan K., Calhoun V.D., Miller L., Stevens M.C., Sahl R., O’Boyle J.G., Schultz R.T., Pearlson G.D. (2010). Abnormal functional connectivity of default mode sub-networks in autism spectrum disorder patients. Neuroimage.

[28] Mann C.A., Lubar J.F., Zimmerman A.W., Miller C.A., Muenchen R.A. (1992). Quantitative analysis of EEG in boys with attention-deficit-hyperactivity disorder: Controlled study with clinical implications. Pediatric Neurol..

[29] Wang J., Barstein J., Ethridge L.E., Mosconi M.W., Takarae Y., Sweeney J.A. (2013). Resting state EEG abnormalities in autism spectrum disorders. J. Neurodev. Disord..

[30] Coben R., Clarke A.R., Hudspeth W., Barry R.J. (2008). EEG power and coherence in autistic spectrum disorder. Clin. Neurophysiol..

[31] Simon D.M., Wallace M.T. (2016). Dysfunction of sensory oscillations in Autism Spectrum Disorder. Neurosci. Biobehav. Rev..

[32] Clarke A.R., Barry R.J., McCarthy R., Selikowitz M. (2001). Age and sex effects in the EEG: Development of the normal child. Clin. Neurophysiol..

[33] Dykman R.A., Holcomb P.J., Oglesby D.M., Ackerman P.T. (1982). Electrocortical frequencies in hyperactive, learning-disabled, mixed, and normal children. Biol. Psychiatry.

[34] Katada A., Ozaki H., Suzuki H., Suhara K. (1981). Developmental characteristics of normal and mentally retarded children’s EEGs. Electroencephalogr. Clin. Neurophysiol..

[35] Stroganova T.A., Nygren G., Tsetlin M.M., Posikera I.N., Gillberg C., Elam M., Orekhova E.V. (2007). Abnormal EEG lateralization in boys with autism. Clin. Neurophysiol..

[36] Spaak E., de Lange F.P., Jensen O. (2014). Local entrainment of alpha oscillations by visual stimuli causes cyclic modulation of perception. J. Neurosci..

[37] Murphy J.W., Foxe J.J., Peters J.B., Molholm S. (2014). Susceptibility to distraction in autism spectrum disorder: Probing the integrity of oscillatory alpha-band suppression mechanisms. Autism Res..

[38] Klimesch W., Sauseng P., Hanslmayr S. (2007). EEG alpha oscillations: The inhibition–timing hypothesis. Brain Res. Rev..

[39] Mathewson K.E., Lleras A., Beck D.M., Fabiani M., Ro T., Gratton G. (2011). Pulsed out of awareness: EEG alpha oscillations represent a pulsed-inhibition of ongoing cortical processing. Front. Psychol..

[40] Chan A.S., Leung W.W.M. (2006). Differentiating autistic children with quantitative encephalography: A 3-month longitudinal study. J. Child Neurol..

[41] McCarty M.J., Brumback A.C. (2021). Rethinking Stereotypies in Autism. Semin. Pediatr. Neurol..

[42] Orekhova E.V., Stroganova T.A., Nygren G., Tsetlin M.M., Posikera I.N., Gillberg C., Elam M. (2007). Excess of high frequency electroencephalogram oscillations in boys with autism. Biol. Psychiatry.

[43] Sutton S.K., Burnette C.P., Mundy P.C., Meyer J., Vaughan A., Sanders C., Yale M. (2005). Resting cortical brain activity and social behavior in higher functioning children with autism. J. Child Psychol. Psychiatry.

[44] Burnette C.P., Henderson H.A., Inge A.P., Zahka N.E., Schwartz C.B., Mundy P.C. (2011). Anterior EEG asymmetry and the modifier model of autism. J. Autism Dev. Disord..

[45] Brugger P., Monsch A.U., Johnson S.A. (1996). Repetitive behavior and repetition avoidance: The role of the right hemisphere. J. Psychiatry Neurosci..

[46] Milovanovic M., Grujicic R. (2021). Electroencephalography in Assessment of Autism Spectrum Disorders: A Review. Front. Psychiatry.

[47] Newson J.J., Thiagarajan T.C. (2019). EEG frequency bands in psychiatric disorders: A review of resting state studies. Front. Hum. Neurosci..

[48] Neuhaus E., Lowry S.J., Santhosh M., Kresse A., Edwards L.A., Keller J., Libsack E.J., Kang V.Y., Naples A., Jack A. (2021). Resting state EEG in youth with ASD: Age, sex, and relation to phenotype. J. Neurodev. Disord..

[49] Casanova M.F., Buxhoeveden D.P., Switala A.E., Roy E. (2002). Minicolumnar pathology in autism. Neurology.

[50] Zafeiriou D.I., Ververi A., Dafoulis V., Kalyva E., Vargiami E. (2013). Autism spectrum disorders: The quest for genetic syndromes. Am. J. Med. Genet. Part B Neuropsychiatr. Genet..

[51] Schwartz S., Kessler R., Gaughan T., Buckley A.W. (2017). Electroencephalogram coherence patterns in autism: An updated review. Pediatric Neurol..

[52] Hoshi H., Shigihara Y. (2020). Age-and gender-specific characteristics of the resting-state brain activity: A magnetoencephalography study. Aging.

